# Expression of metalloproteinases and their inhibitors in primary pulmonary carcinomas.

**DOI:** 10.1038/bjc.1992.434

**Published:** 1992-12

**Authors:** S. J. Urbanski, D. R. Edwards, A. Maitland, K. J. Leco, A. Watson, A. E. Kossakowska

**Affiliations:** Department of Pathology, University of Calgary, Alberta, Canada.

## Abstract

**Images:**


*Br. J. Cancer* (1992), **66**, 1188–1194 © Macmillan Press Ltd., 1992

### Expression of metalloproteinases and their inhibitors in primary pulmonary carcinomas

S.J. Urbanski[1], D.R. Edwards[2], A. Maitland[3], K.J. Leco[2], A. Watson[4] & A.E. Kossakowska[1]

[1]*Department of Pathology, The University of Calgary and Foothills Hospital, 1403–29 Street N.W., Calgary, Alberta T2N 2T9, Canada;* [2]*Department of Pharmacology and Therapeutics, Heritage Medical Research Building, The University of Calgary, 3330 Hospital Drive N.W., Calgary, Alberta T2N 4N1, Canada;* [3]*Department of Surgery, The University of Calgary and Foothills Hospital, 1403–29 Street N.W., Calgary, Alberta T2N 2T9, Canada;* [4]*Department of Medical Biochemistry, The University of Calgary, 2500 University Drive N.W., Calgary, Alberta, T2N 1N4, Canada.*

**Summary** Nine primary pulmonary carcinomas, one metastatic carcinoma, and two malignant pleural mesotheliomas have been analysed for the expression at the mRNA level of metalloproteinases (MPs) and tissue inhibitors of MPs (TIMPs). *In situ* hybridisation showed TIMP-1 and TIMP-2 transcripts predominantly over tumour stroma and gelatinases evenly distributed over both stromal and tumour cells. While both TIMP-1 and TIMP-2 were expressed in non-neoplastic lungs (NNL) as well as in carcinomas, stromelysin 3 (ST3), 92 kDa gelatinase and interstitial collagenase were expressed only by carcinomas. Expression of these MPs by carcinomas was independent of histologic type and such tumour features as fibrosis or necrosis. The consistent expression of ST3 by all of the carcinomas examined and absence of its expression in NNL indicates that ST3 production is likely associated with the malignant phenotype. However, since 92 kDa gelatinase and interstitial collagenase transcripts were found in some but not all tumour samples, their expression is not a uniform feature of pulmonary carcinomas. The possible prognostic significance of the expression of the latter two enzymes by carcinomas remains to be established.

Turnover of the extracellular matrix (ECM) takes place relatively slowly in mature tissues but the pace is greatly accelerated during the tissue remodelling that accompanies processes such as inflammation or malignancy. Matrix degradation is a tightly regulated process in which secreted enzymes of the metalloproteinase (MP) family play key roles (Murphy & Reynolds, 1985; Matrisian, 1991; Birkedal-Hansen *et al.*, 1992). MPs are Zn-dependent proteinases which include interstitial (or specific) collagenase, gelatinases (type IV collagenases), stromelysins (or proteoglycanases) and PUMP-1 (originally, putative metalloproteinase-1) (Whitham *et al.*, 1986; Collier *et al.*, 1988; Muller *et al.*, 1988; Quantin *et al.*, 1989; Wilhelm *et al.*, 1989; Brown *et al.*, 1990). These enzymes display characteristic substrate specificities and in combination they are able to destroy all the constituent proteins of the ECM (Murphy & Reynolds, 1985; Collier *et al.*, 1988; Matrisian, 1991). The function of MPs is regulated at several levels. Transcription of some members of the family is strongly influenced by cytokines and inflammatory mediators (Saklatvala, 1985; Stetler-Stevenson *et al.*, 1990; Circola *et al.*, 1991). Moreover, MPs are released from cells as inactive proenzymes that have to be activated by proteolysis, often by serine proteinases that function in a 'cascade' mechanism (Murphy & Reynolds, 1985; Matrisian, 1991; Birkedal-Hansen *et al.*, 1992). When activated their enzymatic activity is controlled by specific tissue inhibitors (TIMPs). Two TIMPs (TIMP-1 and TIMP-2) have been described both of which have similar activities against the major MPs (Murphy & Reynolds, 1985; Goldberg *et al.*, 1989; Stetler-Stevenson *et al.*, 1989; Boone *et al.*, 1990; Matrisian, 1991; Birkedal-Hansen *et al.*, 1992). Considerable evidence supports the notion that the balance between the levels of extracellular MPs and TIMPs is the primary determinant of the rate of ECM turnover.

The purpose of this study was to investigate the patterns of expression of major MPs and TIMPs in primary lung carcinomas. Correlation of such results with data obtained from non-neoplastic lung tissue (NNL) will identify those MPs which may play significant roles in the pathophysiology of pulmonary carcinomas. It will also help to focus future research on possible associations between these ECM-regulating functions and other factors such as cytokines, the interplay between which may have important consequences for the processes of invasion and metastasis.

Correspondence: Dr S.J. Urbanski, Department of Histopathology, Foothills Hospital, 1403–29th Street N.W., Calgary, Alberta T2N 2T9, Canada.
Received 26 February 1992; and in revised from 27 July 1992.

### Materials and methods

### *Source of tissue*

The tissue was obtained from 10 lungs resected for carcinomas (nine cases of primary carcinomas, one of metastatic colonic carcinoma) and two cases of malignant pleural mesotheliomas. These originated from five women and seven men. Lung resections were received fresh in the Department of Pathology where appropriate sections were taken for RNA extraction, quick frozen in liquid nitrogen and then stored at −70°C. In each case tissue was taken from two aspects of the tumour, one underneath the pleura and one from the opposite aspect (medial). Samples of non-neoplastic lung were taken from sites away from the tumour as controls. The sections of areas taken as controls were examined histologically in order to eliminate tissue which would contain endogenous pneumonia or fibrosis. An additional section of tumour was frozen in liquid nitrogen in OCT-medium and stored at −70°C for subsequent *in situ* hybridisation studies.

### *DNA probes*

Full-length human TIMP-1 and human interstitial collagenase cDNA probes were described previously (Edwards *et al.*, 1987; Kossakowska *et al.*, 1991). The TIMP-2 full length human gene was isolated by polymerase chain reaction (PCR) amplification from cDNA prepared from human MRC5 fetal lung fibroblasts. The PCR product was generated using oligonucleotides that introduced Sma I and Stu I sites 5′ and 3′ of the coding region, giving rise to a 680 bp fragment containing the entire human TIMP-2 coding region (Boone *et al.*, 1990). After digestion with Sma I and Stu I, the PCR product was cloned into EcoRV cut pBluescriptII KS and its identity was confirmed by DNA sequence analysis.

The 72 kDa type IV collagenase was a 210 bp fragment of C-terminal and 3′-non-coding sequences kindly provided by Dr A. Docherty (Celltech, UK), and was also described previously (Kossakowska *et al.*, 1991). The 92 kDa type IV

collagenase probe was a donation from Dr G. Goldberg, Division of Dermatology, Washington University School of Medicine, St. Louis, Missouri, USA (Wilhelm *et al.*, 1989). The stromelysin-3 cDNA probe was a generous gift from Dr P. Chambon, Laboratoire de Génétique Moléculare des Eukaryotes du CNRS, Strasbourg Cedex, France (Basset *et al.*, 1990), and PUMP-1 was obtained from Dr L. Matrisian, Vanderbilt University Medical School, Nashville, Tennesse (Matrisian, 1991).

*RNA isolation and northern blot analysis*

Total cellular RNA was extracted from lung tissue homogenised in guanidinium isothiocyanate and centrifuged through cesium chloride gradients as previously described (Kossakowska *et al.*, 1991). Total cellular RNA (10 µg) from each case was electrophoresed in formaldehyde containing 1.1% agarose gels, transferred to HYBOND-N membranes (Amersham) in 20 × SSC buffer and fixed by baking at 80°C for 2 h. The blots were probed with nick-translated $^{32}$P-labelled DNA probes (specific activity > $10^8$ c.p.m. $\mu g^{-1}$) and autoradiographed using Kodak XAR-5 film (Eastman Kodak, Rochester, NY). Subsequently, blots were reprobed with a murine 18S rRNA probe or stained with methylene blue to confirm equivalence of loading. Comparisons between different blots were made possible by the inclusion of two common samples on each.

*RNA probe preparation and* in situ *hybridisation*

Sense and anti-sense $^{35}$S-RNA probes were prepared from human TIMP-1, TIMP-2, 72 kDa gelatinase and 92 kDa gelatinase cDNAs cloned in either pBluescript KS$^-$ (Stratagene) or pGEM2 (Promega) vectors. Details of the TIMP-1 probes have been described previously (Kossakowska *et al.*, 1991). For TIMP-2 in pBluescript KS$^-$, sense probes were generated by T3 polymerase following digestion with EcoRI, and anti-sense probes were made by T7 polymerase after *Hind*III digestion of template. The sense and anti-sense 72 kDa gelatinase probes were produced from template digested with *Bam*HI and *Xba*I, using T7 and T3 polymerases, respectively. For *in situ* studies a fragment of 92 kDa gelatinase from the 5′ end of the cDNA to a *Bam*HI site at nucleotide 390 (Wilhelm *et al.*, 1989) was subcloned into pBluescript KS$^-$, and sense and anti-sense probes were made from *Not*I and *Eco*RV restricted templates, with T3 and T7 polymerases, respectively.

For *in situ* hybridisation 2–3 µm thick cryostat sections were placed on glass slides coated with 2% aminopropyltriethoxysilane and fixed with 4% para-formaldehyde. After rehydration the sections were washed in 4 × SSC prior to acetylation and pre-hybridisation in 4 × SSPE (Sambrook *et al.*, 1989), 1 × Denhardt's solution, and 50% deionised formamide, 20 mM dithiothreitol and 40 $\mu g\ ml^{-1}$ *E. coli* tRNA at 50°C for 3 h. The labelled riboprobes were diluted in the prehybridisation mixture at a concentration of 3–5 ng of RNA (1–2 × $10^6$ c.p.m.) per 20 µl aliquot per slide. The hybridisation was carried out at 50°C for 18 h. The slides were then processed through the following stringency washes: 2 × SSC at room temperature, 2 × SSC at 50°C, RNase A (4 $\mu g\ ml^{-1}$) treatment for 30 min at 37°C, 2 × SSC at 50°C. Following dehydration through ethanol series the slides were air dried and dipped in Kodak NTB-2 photographic emulsion diluted 1:1 with distilled water. After 2–4 weeks

**Figure 1** Stromelysin-3, TIMP-1, and TIMP-2 expression in lung carcinomas, adjacent non-neoplastic lung tissue, and mesothelioma. All lanes contained total cellular RNA (10 µg) derived from the following cases (as in Table I): lanes 1 to 5, case 9; lane 6, mesothelioma; lanes 7 and 8, case 5; lane 9 and 10, case 8, lane 11, case 2; lanes 12 and 13, case 1. The upper panel represents the blot hybridised with $^{32}$P nick-translated stromelysin-3 DNA probe, the middle panels show the same blot rehybridised with TIMP-1 and TIMP-2 DNA probes and the lowest panel shows loading control hybridisation with 18S rRNA. These autoradiographs were obtained after 48 h exposure at −70°C with an intensifying screen. The blot hybridised with 18S rRNA was exposed for 24 h at room temperature without the intensifying screen. Tissue origins are indicated as: TUP, tumour-pleural; TUM, tumour-medial; L, non-neoplastic lung; PL, uninvolved pleura; M, mesothelioma.

exposure at 4°C the slides were developed in Dektol (Eastman Kodak Co.) and counterstained with haematoxylin and eosin.

### Results

Northern blot analysis was carried out to establish the expression patterns of the 72 kDa and 92 kDa gelatinases, interstitial collagenase, stromelysin 3, PUMP-1, and TIMPs-1 and -2 in the nine primary lung carcinomas, one metastatic colonic carcinoma to lung, two mesotheliomas, and NNL (including uninvolved pleura). The NNL included seven cases of morphologically normal lung adjacent to carcinomas. Representative data are shown in Figures 1 and 2, and the results are summarised in Table I.

For some of the genes under study, striking differences in expression patterns between RNA isolated from carcinomas and NNL were observed. Transcripts corresponding to ST3, 92 kDa gelatinase and interstitial collagenase were found only in tumour samples, but not in NNL. For ST3, RNA levels were low in all but two primary adenocarcinomas, where strong signals were obtained from samples from the pleural tumour aspect. Metastatic colonic adenocarcinoma and mesotheliomas also expressed ST3 RNA. In the case of 92 kDa gelatinase, it was apparent that it was not always expressed in the same tumours as ST3, or in the same locations (i.e. medial *vs* pleural). Transcripts for 92 kDa gelatinase were present in five primary and one metastatic carcinoma while no signal was seen in the remaining four tumours. In most of the five positive cases, the signals were relatively low with no appreciable difference in levels between pleural and medial tumour aspects, except in the case of the metastatic colonic carcinoma in which transcripts were not detected in the medial aspect. Interstitial collagenase RNA transcripts were detected in three out of nine carcinomas analysed.

The RNAs coding for TIMP-1 and TIMP-2, 72 kDa gelatinase and PUMP-1 were present in malignant samples as well as NNL. Our experiences with many Northern blots suggest that TIMP-1 is the most highly expressed of all of the genes that we have analysed but its expression is variable and generally higher in tumour *vs* non-neoplastic lung samples (Figure 1). As reported previously, we observed two major classes of TIMP-2 transcripts of 3.5 kb and 1.0 kb sizes, with an additional minor 2.5 kb form in most tissues (Figure 1) (Leco *et al.*, 1992; Stetler-Stevenson *et al.*, 1990). In almost all cases the 3.5 kb species was the most abundant with the 1.0 kb form being barely visible for some tumour RNAs, as has been seen previously in RNAs extracted from colorectal tumours (Stetler-Stevenson *et al.*, 1990).

Two additional points are apparent from inspection of the data presented in Table I and Figures 1 and 2. Firstly, both malignant pleural mesotheliomas studied showed no expression of 72 kDa and 92 kDa gelatinases and PUMP-1, whereas the majority of carcinomas expressed these genes. Secondly, all analysed adenocarcinomas exhibited some degree of fibrosis, while necrosis was more prominent in

**Figure 2** Expression of RNAs encoding 72 kDa and 92 kDa gelatinases, interstitial collagenase and PUMP-1 in human lung carcinomas, adjacent non-neoplastic lung tissue, and mesotheliomas. All lanes contained total cellular RNA (10 μg) derived from the following cases (as in Table I): lane 1, mesothelioma (the same case as lane 6 from Figure 1); lanes 2 and 3, case 9; lanes 4 to 7, case 2; lane 8, second mesothelioma; lanes 9 to 12, case 8. The upper panels represent Northern blots hybridised with 72 kDa and 92 kDa gelatinases, interstitial collagenase and PUMP-1 $^{32}P$ nick-translated DNA probes and the lowest panel displays the same blot rehybridised with 18S rRNA probe (loading control). The hybridised blots have been exposed for 48 h at −70°C with an intensifying screen. The blot hybridised with 18S rRNA was exposed for 24 h at room temperature without the intensifying screen.

**Table I** Summary of Northern blot results of lung carcinomas extracts probes for 72 kDa and 92 kDa gelatinase, TIMP-1 and TIMP-2, interstitial collagenase, stromelysin 3 and PUMP-1

| *Case No. and tumour aspect* | *Lobe and laterality* | *Diagnosis* | *TIMP-1* | *TIMP-2* | *72 kDa* | *92 kDa* | *Interstitial collagenase* | *Stromelysin 3* | *PUMP-1* | *Age* | *Sex* | *Pleural invasion* | *Fibrosis* | *Necrosis* |
|---|---|---|---|---|---|---|---|---|---|---|---|---|---|---|
| 1. M | RUL | Adenocarcinoma | +++ | + | – | – | n/a | + | n/a | 75 | M | + | +++ | – |
| P | | | ++ | ++ | ++ | – | n/a | – | n/a | | | | | |
| PL | | | +++ | ++ | – | – | n/a | – | n/a | | | | | |
| 2. M | LLL | Squamous cell carcinoma | ++ | + | – | + | – | ++ | + | 53 | M | – | ++ | ++ |
| P | | | + | + | – | – | – | – | – | | | | | |
| L | | | + | + | + | – | – | – | + | | | | | |
| 3. M | RUL | Squamous cell carcinoma | +++ | + | + | – | – | n/a | + | 72 | M | + | + | +++ |
| P | | | +++ | ++ | – | – | + | ++ | ++ | | | | | |
| 4. M | LLL | Metastatic colonic adenocarcinoma | ++ | + | + | – | – | + | n/a | 60 | F | – | – | – |
| P | | | +++ | + | – | + | – | + | n/a | | | | | |
| PL | | | n/a | ++ | + | – | – | – | n/a | | | | | |
| 5. M | RLL | Small cell carcinoma | – | + | – | + | – | + | – | 72 | F | – | – | + |
| P | | | + | + | – | + | – | + | + | | | | | |
| PL | | | n/a/ | ++ | + | – | + | – | n/a | | | | | |
| 6. M | LUL | Adenocarcinoma | + | + | + | – | – | + | + | 63 | F | + | + | – |
| P | | | + | + | + | – | – | + | – | | | | | |
| 7. M | RLL | Adenocarcinoma | ++ | ++ | – | – | – | + | + | 72 | F | + | + | + |
| P | | | + | ++ | – | – | – | + | + | | | | | |
| 8. M | RUL | Adenocarcinoma | ++ | ++ | + | + | – | + | + | 66 | M | + | + | + |
| P | | | ++ | ++ | + | + | – | +++ | + | | | | | |
| L | | | + | + | + | – | – | – | + | | | | | |
| 9. M | RUL | Adenocarcinoma | +++ | ++ | + | ++ | + | ++ | +++ | 70 | F | – | + | + |
| P | | | +++ | ++ | + | +++ | +++ | +++ | ++ | | | | | |
| L | | | + | ++ | + | – | – | – | + | | | | | |
| PL | | | + | ++ | + | – | – | – | + | | | | | |
| 10. M | RUL | Squamous cell carcinoma | + | + | + | + | – | + | + | 65 | M | – | – | +++ |
| P | | | + | + | + | + | – | + | + | | | | | |
| L | | | + | + | + | – | – | – | + | | | | | |

M, medial; P, pleural; L, morphologically normal lung adjacent to tumour; PL, uninvolved pleura; RUL, right upper lobe; LUL, left upper lobe; RLL, right lower lobe; LLL, left lower lobe; n/a, not analysed. The intensity of signal is subjectively expressed as undetectable (–), weak (+), moderate (++), or strong (+++).

**Figure 3** *In situ* hybridisation of squamous cell carcinoma with TIMP-1 (A1) and TIMP-2 (B1) $^{35}S$ rUTP-labelled anti-sense RNA probes demonstrated signal predominantly over tumour stromal cells in both instances. A2 and B2 represent corresponding sense controls (H&E counterstain × 160).

squamous cell carcinomas. However, neither of these morphologic parameters showed a consistent association with the expression patterns of particular MPs or TIMPs.

We carried out *in situ* hybridisation to tumour sections in order to localise transcripts corresponding to TIMP-1, TIMP-2, 72 kDa, and 92 kDa gelatinases. Differences in the sites of expression of MPs and TIMPs were apparent, with anti-sense TIMP-1 and TIMP-2 probes generating signals predominantly over stromal elements (Figure 3, A1 and B1), whereas anti-sense probes for both of the gelatinases resulted in signals that were evenly distributed over both tumour and stromal cells (Figure 4, C1 and D1). Control sense-strand RNA probes demonstrated that the signals observed were specific (Figures 3 and 4, A2, B2, C2, D2); in addition, pre-treatment with RNase A ($4\,\mu g\,ml^{-1}$) eliminated the hybridisation obtained with all of the anti-sense RNA probes (data not shown).

### Discussion

We undertook the studies reported here in order to determine whether different histologic types of lung carcinomas showed characteristic patterns of expression of matrix remodelling enzymes and inhibitors that have been linked with tumour cell invasion. Moreover, we wished to know whether invasion *in vivo* on the medial and pleural fronts of carcinomas might represent specific remodelling phenomena that call for localised production of particular degradative enzymes. Our data reveal that certain MPs are frequently observed to be expressed at elevated levels in carcinomas, but expression of none of the genes analysed to date could be correlated with a particular tumour type or part of tumour (medial *vs* pleural).

Based on the analysis of a collection of nine primary pulmonary carcinomas, we have detected not only differences in the expression of MPs between carcinomas and NNL but also heterogeneity in the expression of MPs among the tumours. Expression of three MPs – ST3, 92 kDa gelatinase, and interstitial collagenase – was detected in carcinomas but not in NNL. ST3 was previously observed in 30 breast carcinomas but only in low levels in one of five fibroadenomas (Basset *et al.*, 1990). Detection of the same pattern of expression in lung carcinomas supports the notion that ST3 represents an enzyme that is principally associated with the malignant phenotype. ST3 expression characterises not only primary carcinomas as it was also detected in pulmonary metastases from colonic carcinoma. It has been also previously noted that 92 kDa gelatinase RNA was seen primarily in breast carcinomas but not in fibroadenomas (Basset *et al.*, 1990). However, not all breast carcinomas expressed this proteinase (Basset *et al.*, 1990), and we demonstrate that this situation also holds for human lungs where 92 kDa gelatinase was expressed exclusively by carcinomas but only in five out of eight analysed primary tumours. Metastatic colonic adenocarcinoma also contained 92 kDa gelatinase RNAs. *In situ* hybridisation revealed that 92 kDa gelatinase transcripts were evenly distributed between stromal and tumour cells. Histiocytes, which are known to produce 92 kDa gelatinase (Wilhelm *et al.*, 1989), were not abundant in the tumour sections, indicating that the total contribution of these cells to the levels of 92 kDa gelatinase transcripts in lung carcinomas must be small.

The third MP whose expression was linked to the malignant phenotype was interstitial collagenase, transcripts for which were seen in three out of eight carcinomas, but not in the metastatic carcinomas or in any sample of NNL. Inspec-

**Figure 4** *In situ* hybridisation of squamous cell carcinoma with $^{35}$S-labelled anti-sense RNA probe corresonding to 72 kDa (C1) and 92 kDa (D1) gelatinases demonstrated signal over tumour cells and stromal cells in both instances. C2 and D2 represent corresponding sense controls (H&E counterstain × 160).

tion of Table I shows however, that 92 kDa gelatinase, interstitial collagenase and ST3 are expressed independently of each other and that their patterns of expression are not influenced by or linked with such tumour features as the amount of fibrosis or necrosis.

Both TIMP genes and PUMP-1 were expressed in all normal and malignant tissue samples analysed. The TIMP-2 signal, which is largely attributable to the 3.5 kb RNA, was relatively constant in both neoplastic and non-neoplastic lung samples. In agreement with previous studies (Stetler-Stevenson *et al.*, 1990; Kossakowska *et al.*, 1991), our data demonstrate that TIMP-1 is expressed predominantly by host stromal cells with transcript levels being elevated in some of the tumour samples, possibly indicating the influence of tumour-derived diffusible factors. Our data are the first to demonstrate spatially-restricted TIMP-2 expression to stromal elements in tumour. These findings support the idea that the TIMPs may play a role in human neoplasia due to their abilities to inhibit the active forms of MPs (DeClerck *et al.*, 1991).

In summary, our conclusions are:

1. Stromelysin 3 is consistently found to be expressed in carcinomas but not in non-neoplastic lung tissue.
2. 92 kDa gelatinase and interstitial collagenase transcripts are also absent from NNL and are present in some but not all carcinomas.
3. Transcripts for 92 kDa and 72 kDa gelatinases are present in both tumour and stromal cells, whereas TIMP-1 and TIMP-2 RNAs are principally localised to host stroma.

This study extends to pulmonary carcinoma the association between expression of ST3 and malignancy that had been made from investigations of breast carcinoma (Basset *et al.*, 1990). It also indicates that 92 kDa gelatinase and interstitial collagenase may play important roles in the biology of certain carcinomas. More cases will be anlaysed and followed for an adequate period of time in order to establish any prognostic significance of expression of these two proteinases.

This work has been supported by grants from the Alberta Cancer Board and Medical Research Council of Canada. D.R. Edwards is an Alberta Heritage Foundation for Medical Research (AHFMR) Scholar, and K.J. Leco is the recipient of an AHFMR graduate studentship.

We are thankful to Alannah Ireland and Betty Hood for the preparation of the manuscript and to Susan Hui for her technical assistance.

### References

BASSET, P., BELLOCQ, J.P., WOLF, C., STOLL, I., HUTIN, P., LIMACHER, J.M., PODHAJCER, O.L., CHENARD, M.P., RIO, M.C. & CHAMBON, P. (1990). A novel metalloproteinase gene specifically expressed in stromal cells of breast carcinomas. *Nature*, **348**, 699–704.

BIRKEDAL-HANSEN, H., WERB, Z., WELGUS, H. & VAN WART, H. (1992). (eds). *Matrix Metalloproteinase and Inhibitors*. Gustav Fischer Verlag: Stuttgart, Jena, New York.

BOONE, T.C., JOHNSON, M.J., DECLERCK, Y.A. & LANGLEY, K.E. (1990). cDNA cloning and expression of a metalloproteinase inhibitor related to tissue inhibitor of metalloproteinases. *Proc. Natl Acad. Sci. USA*, **87**, 2800–2804.

BROWN, P.D., LEVY, A.T., MARGULIES, I.M.K., LIOTTA, L.A. & STETLER-STEVENSON, W.G. (1990). Independent expression and cellular processing of M 72,000 type IV collagenase and interstitial collagenase in human tumorigenic cell lines. *Cancer Res.*, **50**, 6184–6191.

CIRCOLA, A., WELGUS, H.G., PIERCE, G.F., KRAMER, K. & STRUNK, R.C. (1991). Differential regulation of the expression of proteinases/anti-proteinases in fibroblasts. *J. Biol. Chem.*, **266**, 12283–12288.

COLLIER, I.E., WILHELM, S.M., EISEN, A.Z., MARMER, B.L., GRANT, G.A., SELTZER, J.L., KRONBERGER, A., HE, C., BAUER, E.A. & GOLDBERG, G.I. (1988). H-ras oncogene-transformed human bronchial epithelial cells (TBE-1) secrete a single metalloprotease capable of degrading basement membrane collagen. *J. Biol. Chem.*, **263**, 6579–6587.

DECLERCK, Y.A., YEAN, T.D., LU, H.S., TING, J. & LANGLEY, K.E. (1991). Inhibition of autoproteolytic activation of interstitial procollagenase by recombinant metalloproteinase inhibitor MI/TIMP-2. *J. Biol. Chem.*, **266**, 3893–3899.

EDWARDS, D.R., MURPHY, G., REYNOLDS, J.J., WHITHAM, S.E., DOCHERTY, A.J.P., ANGEL, P. & HEATH, J.K. (1987). Transforming growth factor beta modulates the expression of collagenase and metalloproteinase inhibitor. *EMBO J.*, **6**, 1899–1904.

GOLDBERG, G.I., MARMER, B.L., GRANT, G.A., EISEN, A.Z., WILHELM, S. & HE, C. (1989). Human 72-kilodalton type collagenase forms a complex with a tissue inhibitor of metalloproteases designated TIMP-2. *Proc. Natl Acad. Sci. USA*, **86**, 8207–8211.

KOSSAKOWSKA, A.E., URBANSKI, S.J. & EDWARDS, D.R. (1991). Tissue inhibitor of metalloproteinases-1 (TIMP-1) RNA is expressed at elevated levels in malignant non-Hodgkin's lymphomas. *Blood*, **77**, 2475–2481.

LECO, K.J., HAYDEN, L.J., SHARMA, R.R., ROCHELEAU, H., GREENBERG, A.H. & EDWARDS, D.R. (1992). Differential regulation of TIMP-1 and TIMP-2 in normal and ras-transformed murine fibroblasts. *Gene*, **117**, 209–217.

MATRISIAN, L.M. (1991). Metalloproteinases and their inhibitors in matrix modelling. *Trends Genet.*, **6**, 121–125.

MULLER, D., QUANTIN, B., GESNEL, M.-C., MILLON-COLLARD, R., ABECASSIS, J. & BREATHNACH, R. (1988). The collagenase gene family in humans consists of at least four members. *Biochem. J.*, **253**, 187–192.

MURPHY, G. & REYNOLDS, J.J. (1985). Current views of collagen degradation. *Bioessays*, **2**, 55–60.

QUANTIN, B., MURPHY, G. & BREATHNACH, R. (1989). PUMP-1 cDNA codes for a protein with characteristics similar to those of classical collagenase family members. *Biochemistry*, **28**, 5327–5334.

SAKLATVALA, J. (1986). Tumour necrosis factor α stimulates resorption and inhibits synthesis of proteoglycan in cartilage. *Nature*, **322**, 547–549.

SAMBROOK, J., FRITSCH, E.F. & MANIATIS, T. (1989). *Molecular Cloning: A Laboratory Manual.* Cold Spring Harbor Laboratory.

STETLER-STEVENSON, W.G., BROWN, P.D., ONISTO, M., LEVY, A.T. & LIOTTA, L.A. (1990). Tissue inhibition of metalloproteinases-2 (TIMP-2) mRNA expression in tumor cell lines and human tumor tissues. *J. Biol. Chem.*, **265**, 13933–13938.

STETLER-STEVENSON, W.G., KRUTZSCH, H.C. & LIOTTA, L.A. (1989). Tissue inhibitor of metalloproteinase (TIMP-2): A new member of the metalloproteinase inhibitor family. *J. Biol. Chem.*, **264**, 17374–17378.

WHITHAM, S.E., MURPHY, G., ANGEL, P., RAHMSDORF, H.J., SMITH, B.J., LYONS, A., HARRIS, T.J.R., REYNOLDS, J.J., HERRLICH, P. & DOCHERTY, A.J.P. (1986). Comparison of human stromelysin and collagenase by cloning and sequence analysis. *Biochem. J.*, **240**, 913–916.

WILHELM, S.M., COLLIER, I.E., MARMER, B.L., EISEN, A.Z., GRANT, G.A. & GOLDBERG, G.I. (1989). SV40-transformed human lung fibroblasts secrete a 92-kDa type IV collagenase which is identical to that secreted by normal human macrophages. *J. Biol. Chem.*, **264**, 17213–17221.


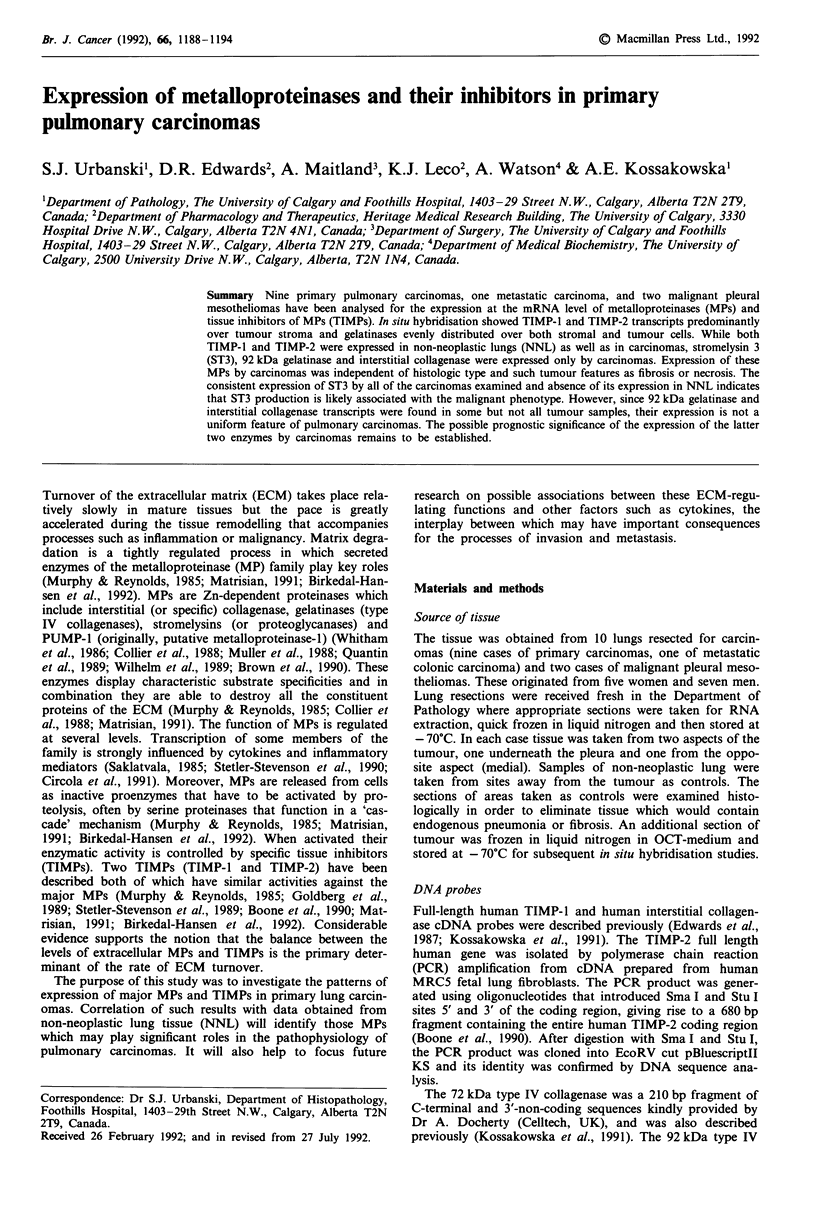

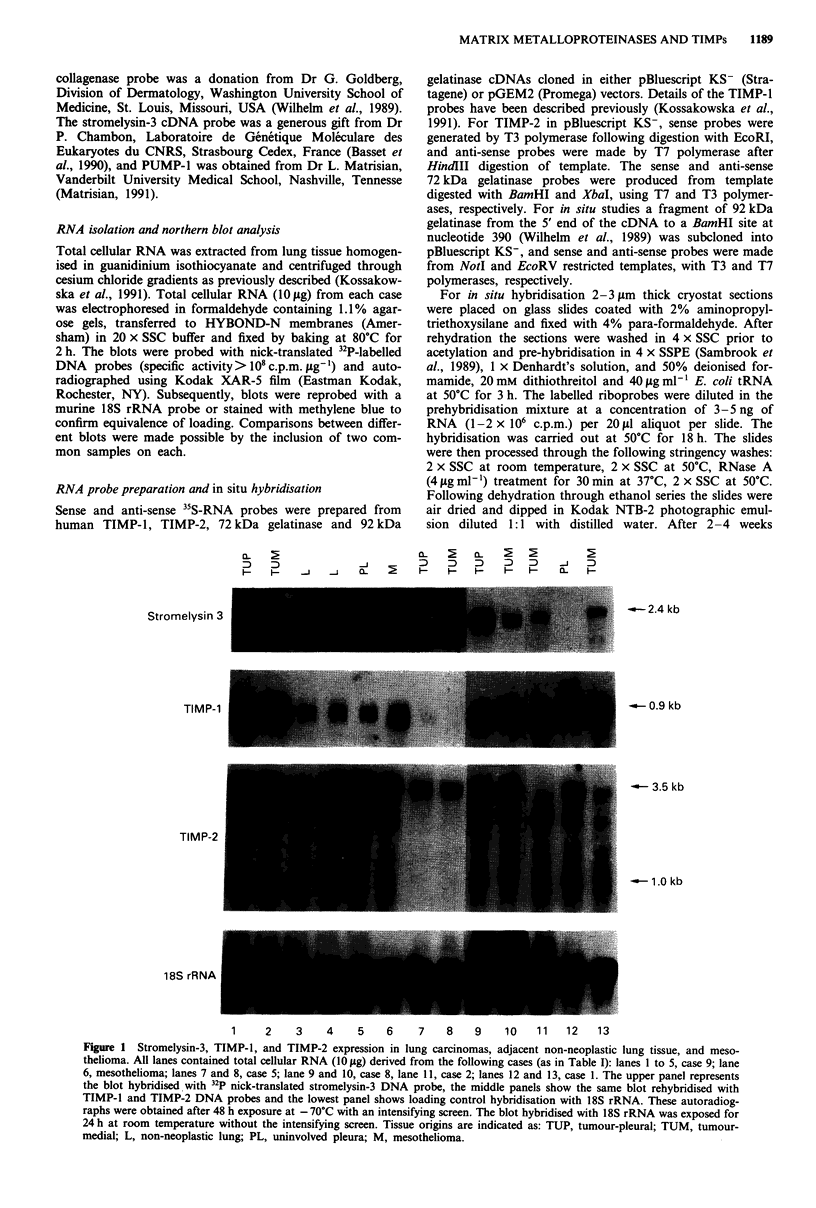

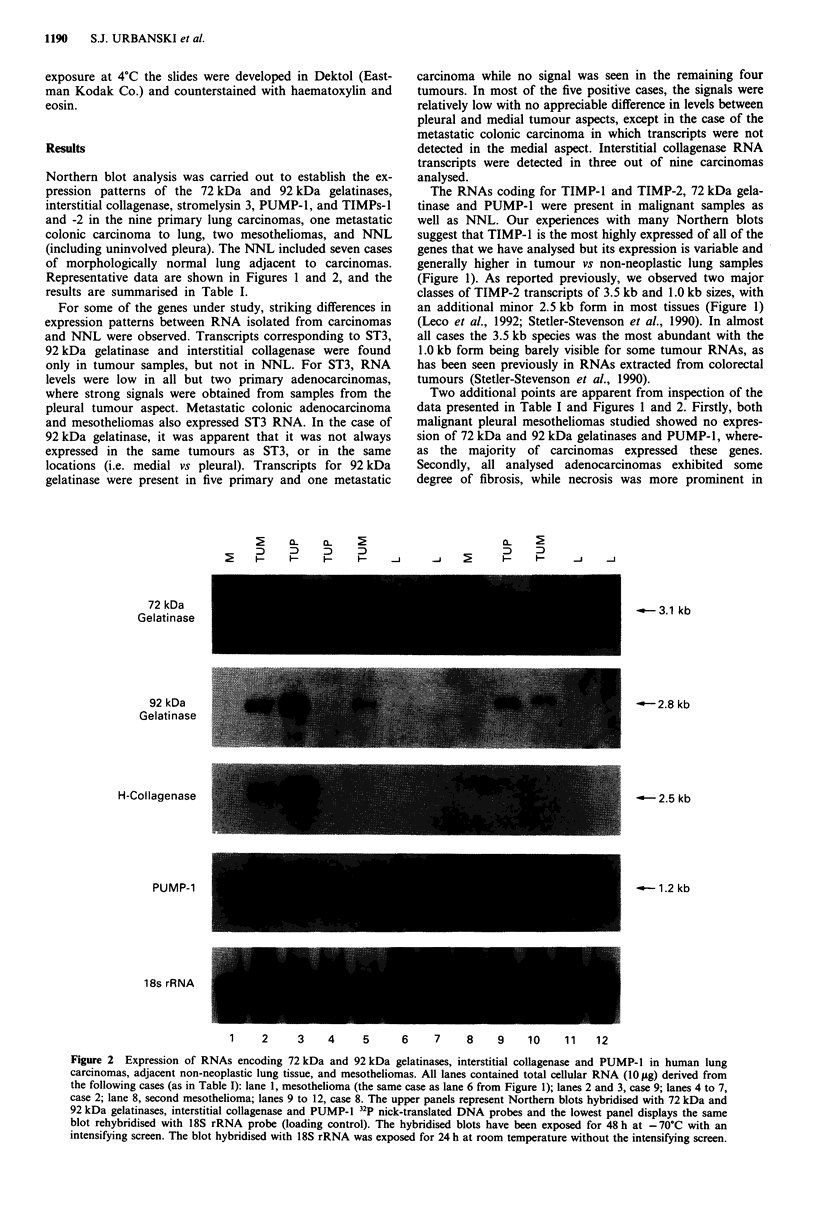

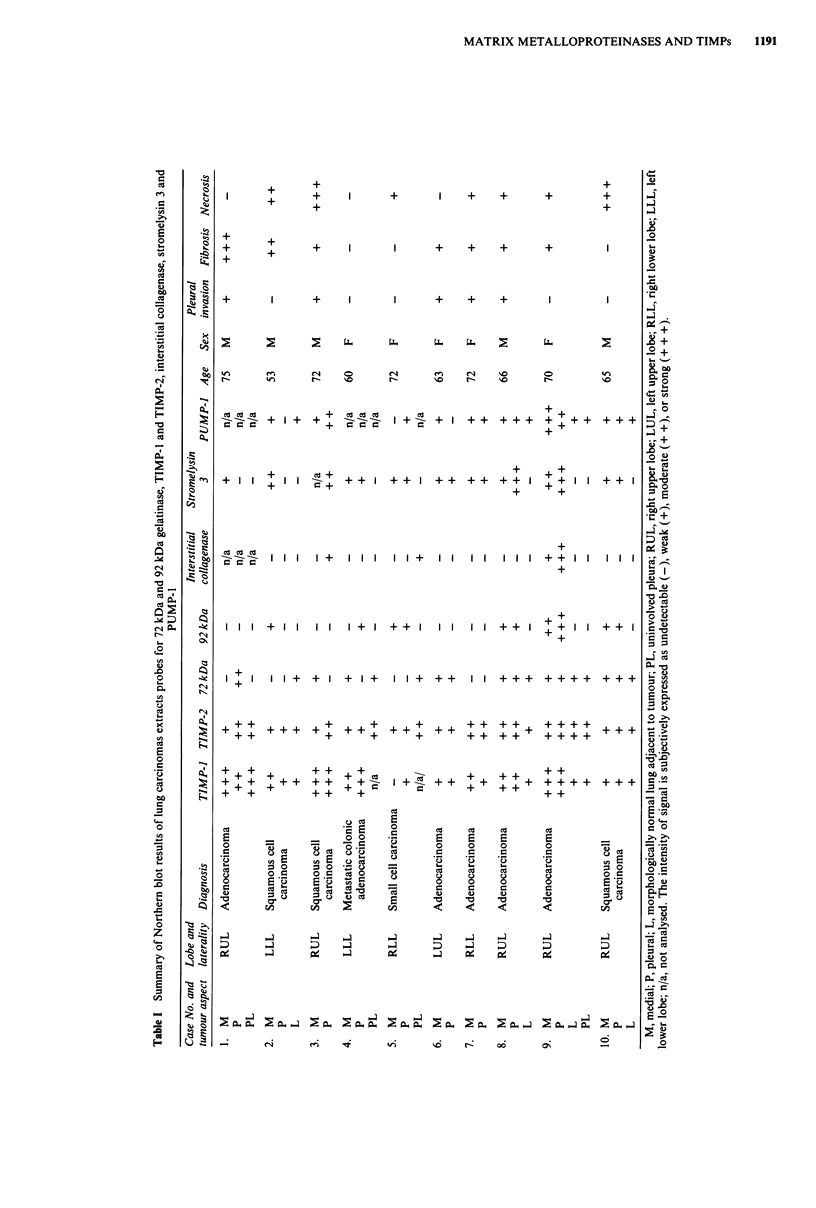

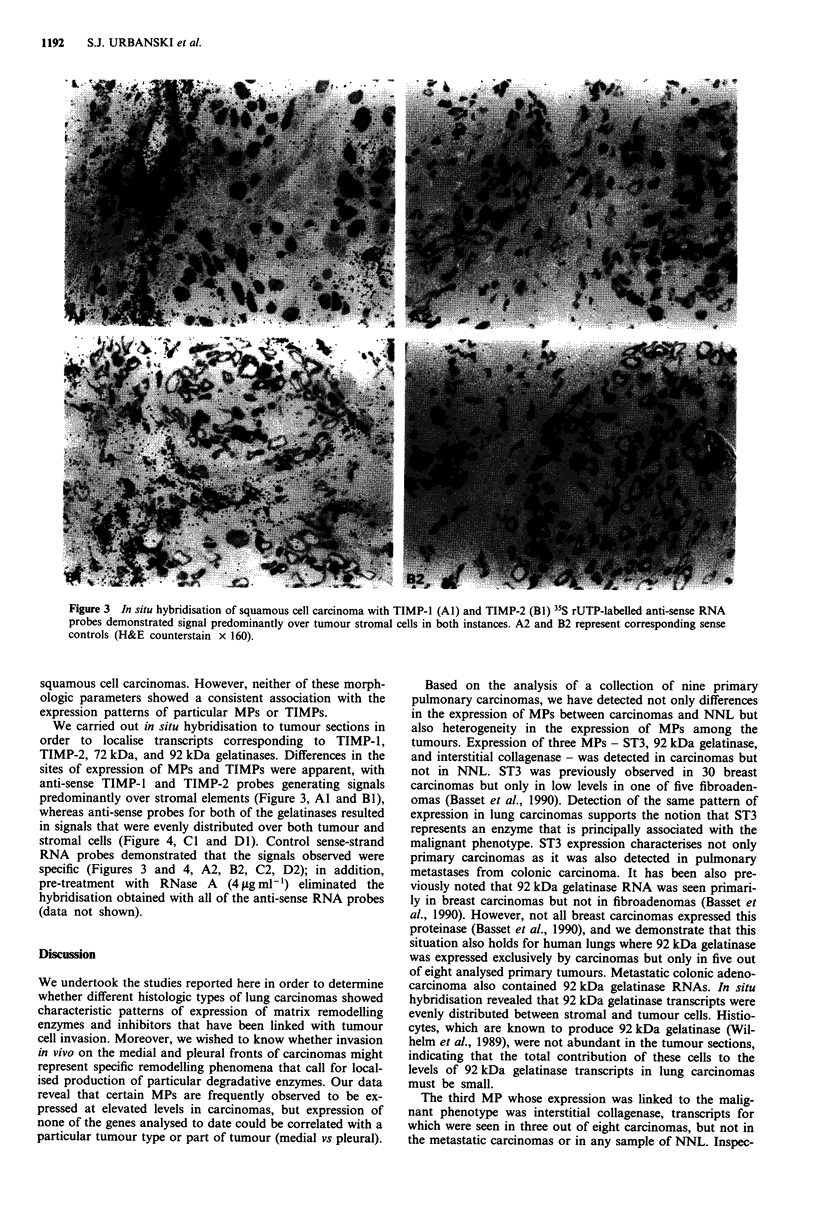

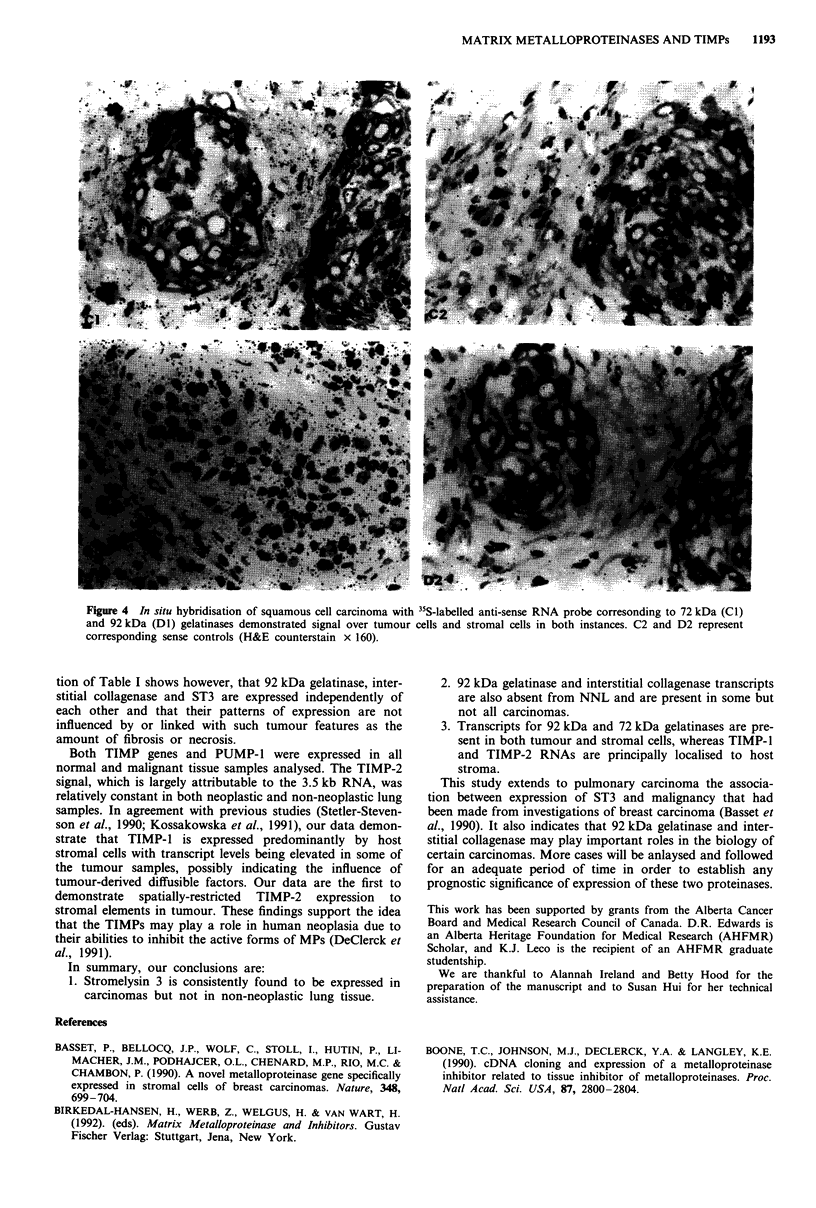

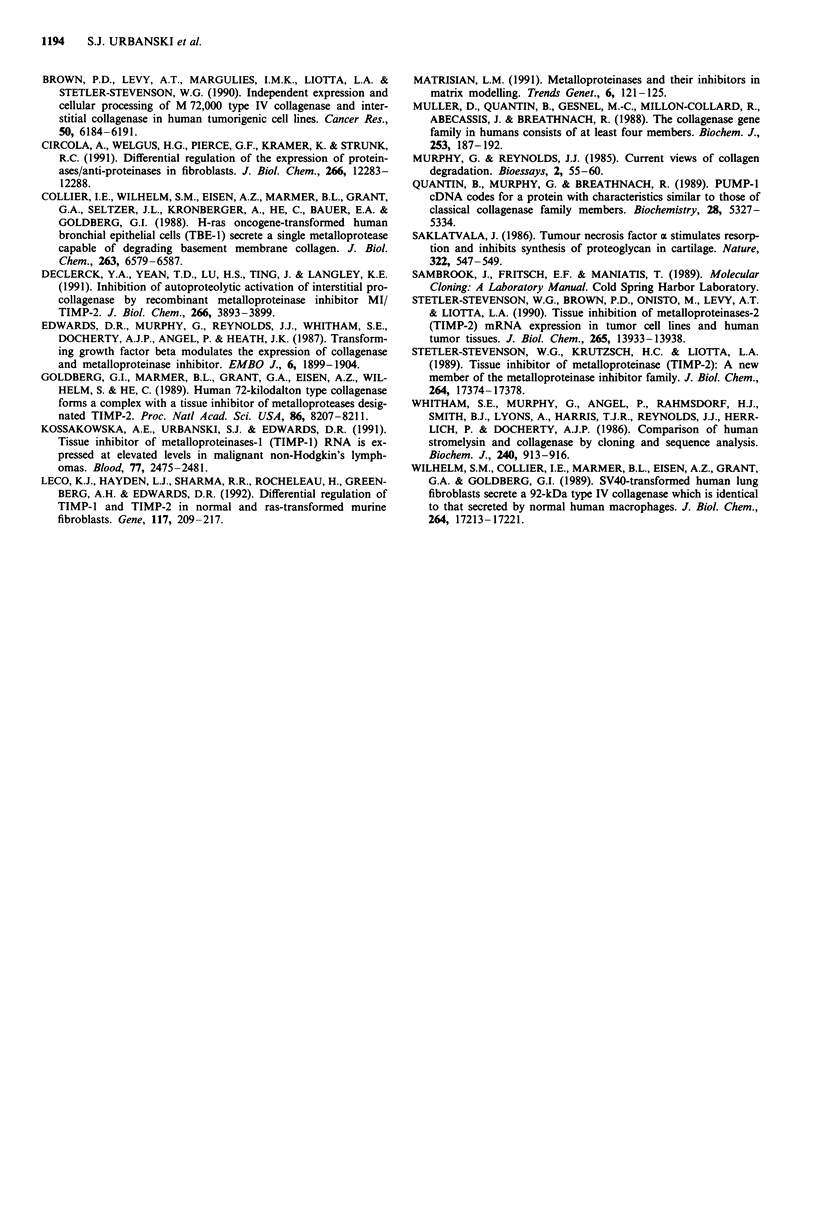


## References

[OCR_00709] Basset P., Bellocq J. P., Wolf C., Stoll I., Hutin P., Limacher J. M., Podhajcer O. L., Chenard M. P., Rio M. C., Chambon P. (1990). A novel metalloproteinase gene specifically expressed in stromal cells of breast carcinomas.. Nature.

[OCR_00719] Boone T. C., Johnson M. J., De Clerck Y. A., Langley K. E. (1990). cDNA cloning and expression of a metalloproteinase inhibitor related to tissue inhibitor of metalloproteinases.. Proc Natl Acad Sci U S A.

[OCR_00727] Brown P. D., Levy A. T., Margulies I. M., Liotta L. A., Stetler-Stevenson W. G. (1990). Independent expression and cellular processing of Mr 72,000 type IV collagenase and interstitial collagenase in human tumorigenic cell lines.. Cancer Res.

[OCR_00734] Circolo A., Welgus H. G., Pierce G. F., Kramer J., Strunk R. C. (1991). Differential regulation of the expression of proteinases/antiproteinases in fibroblasts. Effects of interleukin-1 and platelet-derived growth factor.. J Biol Chem.

[OCR_00740] Collier I. E., Wilhelm S. M., Eisen A. Z., Marmer B. L., Grant G. A., Seltzer J. L., Kronberger A., He C. S., Bauer E. A., Goldberg G. I. (1988). H-ras oncogene-transformed human bronchial epithelial cells (TBE-1) secrete a single metalloprotease capable of degrading basement membrane collagen.. J Biol Chem.

[OCR_00748] DeClerck Y. A., Yean T. D., Lu H. S., Ting J., Langley K. E. (1991). Inhibition of autoproteolytic activation of interstitial procollagenase by recombinant metalloproteinase inhibitor MI/TIMP-2.. J Biol Chem.

[OCR_00754] Edwards D. R., Murphy G., Reynolds J. J., Whitham S. E., Docherty A. J., Angel P., Heath J. K. (1987). Transforming growth factor beta modulates the expression of collagenase and metalloproteinase inhibitor.. EMBO J.

[OCR_00762] Goldberg G. I., Marmer B. L., Grant G. A., Eisen A. Z., Wilhelm S., He C. S. (1989). Human 72-kilodalton type IV collagenase forms a complex with a tissue inhibitor of metalloproteases designated TIMP-2.. Proc Natl Acad Sci U S A.

[OCR_00766] Kossakowska A. E., Urbanski S. J., Edwards D. R. (1991). Tissue inhibitor of metalloproteinases-1 (TIMP-1) RNA is expressed at elevated levels in malignant non-Hodgkin's lymphomas.. Blood.

[OCR_00774] Leco K. J., Hayden L. J., Sharma R. R., Rocheleau H., Greenberg A. H., Edwards D. R. (1992). Differential regulation of TIMP-1 and TIMP-2 mRNA expression in normal and Ha-ras-transformed murine fibroblasts.. Gene.

[OCR_00778] Matrisian L. M. (1990). Metalloproteinases and their inhibitors in matrix remodeling.. Trends Genet.

[OCR_00782] Muller D., Quantin B., Gesnel M. C., Millon-Collard R., Abecassis J., Breathnach R. (1988). The collagenase gene family in humans consists of at least four members.. Biochem J.

[OCR_00792] Quantin B., Murphy G., Breathnach R. (1989). Pump-1 cDNA codes for a protein with characteristics similar to those of classical collagenase family members.. Biochemistry.

[OCR_00798] Saklatvala J. (1986). Tumour necrosis factor alpha stimulates resorption and inhibits synthesis of proteoglycan in cartilage.. Nature.

[OCR_00806] Stetler-Stevenson W. G., Brown P. D., Onisto M., Levy A. T., Liotta L. A. (1990). Tissue inhibitor of metalloproteinases-2 (TIMP-2) mRNA expression in tumor cell lines and human tumor tissues.. J Biol Chem.

[OCR_00812] Stetler-Stevenson W. G., Krutzsch H. C., Liotta L. A. (1989). Tissue inhibitor of metalloproteinase (TIMP-2). A new member of the metalloproteinase inhibitor family.. J Biol Chem.

[OCR_00821] Whitham S. E., Murphy G., Angel P., Rahmsdorf H. J., Smith B. J., Lyons A., Harris T. J., Reynolds J. J., Herrlich P., Docherty A. J. (1986). Comparison of human stromelysin and collagenase by cloning and sequence analysis.. Biochem J.

[OCR_00825] Wilhelm S. M., Collier I. E., Marmer B. L., Eisen A. Z., Grant G. A., Goldberg G. I. (1989). SV40-transformed human lung fibroblasts secrete a 92-kDa type IV collagenase which is identical to that secreted by normal human macrophages.. J Biol Chem.

